# FGSI: distant supervision for relation extraction method based on fine-grained semantic information

**DOI:** 10.1038/s41598-023-41354-4

**Published:** 2023-08-28

**Authors:** Chenghong Sun, Weidong Ji, Guohui Zhou, Hui Guo, Zengxiang Yin, Yuqi Yue

**Affiliations:** College of Computer Science and Information Engineering, Harbin Normal University, Harbin, 150025 China

**Keywords:** Computer science, Information technology

## Abstract

Relation extraction is one of the important steps in building a knowledge graph. Its main objective is to extract semantic relationships from identified entity pairs in sentences, playing a crucial role in semantic understanding and knowledge graph construction. Remote supervised relation extraction aligns knowledge bases with natural language texts and generates labeled data, which alleviates the burden of manually annotating datasets. However, the labeled corpus obtained from remote supervision contains a large amount of noisy data, which greatly affects the training of relation extraction models. In this paper, we propose the hypothesis that key semantic information within the sentence plays a crucial role in entity relation extraction in the task of remote supervised relation extraction. Based on this hypothesis, we divide the sentence into three segments by splitting it according to the positions of entities, starting from within the sentence. Then, using intra-sentence attention mechanisms, we identify fine-grained semantic features within the sentence to reduce the interference of irrelevant noise information. We also improved the intra-bag attention mechanism by setting a threshold gate to filter out low-relevant noisy sentences, minimizing the impact of noise on the relation extraction model, and making full use of available positive semantic information. Experimental results show that the proposed relation extraction model in this paper achieves improvements in precision-recall curve, P@N value, and AUC value compared to existing methods, demonstrating the effectiveness of this model.

## Introduction

Relationship extraction aims to identify the relationship between entity pairs in plain text sentences to obtain structured knowledge information, i.e., triple information (Entity A, Relation, Entity B), which is an important research hotspot in natural language processing^1^ and an essential preparatory work for constructing knowledge graphs^2^. Currently, machine learning methods for relationship extraction can be divided into unsupervised learning^3^, supervised learning^4^, semi-supervised learning^5^, and remote supervision learning^6^ according to whether the required training corpus is annotated. Although supervised learning methods for relationship extraction have high accuracy and satisfactory overall performance, they require manual annotation of the dataset before model training, which involves a significant amount of human, material, and financial resources. With the continuous development of relationship extraction technology, Mintz et al.^6^ proposed the idea of remote supervision in 2009, which automatically aligns the knowledge base with plain text to generate annotated data. The main idea is based on a strong assumption that “if two entities have a certain relationship in the knowledge base, then all sentences containing these two entities will express this relationship.” For example, (Huawei, founder, Ren Zhengfei) is a triple relationship instance in Freebase, and all sentences containing these two entities will be labeled as founder relationship. However, the remote supervision method proposed by Mintz et al.^6^ still has flaws. The strong assumption they proposed for relationship extraction tasks leads to incorrect annotation problems in the generated dataset, resulting in noise interference in the actual model training process and affecting model performance.

One of the main research directions for distant supervision relation extraction is to develop denoising methods for the relation model, as proposed by Yang Suizhu et al.^7^. In recent years, scholars have proposed various solutions for sample denoising. Surdeanu et al.^8^ addressed the noisy label problem by adopting a multi-instance learning strategy. Takamatsu et al.^9^ designed a generative model to identify patterns of positive and negative samples, discarding negative pattern samples and retaining positive pattern samples to improve the overall performance of the relation extraction model. Zeng et al.^10^ considered the limitations of traditional natural language processing tools and proposed the use of convolutional neural networks for relation extraction, using word vectors and word position vectors as inputs, which achieved better results than classical machine learning models. Nguyen et al.^11^ proposed using windows of multiple scales to extract multidimensional features instead of conventional lexical features, which achieved better results than traditional convolutional neural network models. Zeng et al.^12^ designed a segmented convolutional neural network to extract sentence features and used multi-instance learning to eliminate annotation errors in incorrect samples, reducing the impact of erroneous samples on the overall model performance. Yan Xu et al.^13^ first proposed using Long Short-Term Memory (LSTM) networks for relation extraction and extracted key information through the shortest dependency path, enabling better extraction of sentence-level relations. Lin et al.^14^ improved the selection of training sentences in each bag of multi-instance learning by designing a bag-level attention mechanism to score all sentences in the bag and integrate all sentence information for relation extraction, achieving better results than the baseline model. Ji et al.^15^ introduced entity description information and sentence-level attention mechanism for distant supervision relation extraction, further enriching entity information, reducing noise interference, and achieving better results than previous baseline models. Zhou et al.^16^ proposed using hierarchical selective attention for distant supervision relation extraction, where coarse sentence-level attention was used to select relevant sentences, word-level attention was used to construct sentence representations, and fine-grained sentence-level attention was used to aggregate sentence representations as model inputs, demonstrating the superior performance of their model through experiments. Jianzhou et al.^17^ proposed an improved attention mechanism for relation extraction, in which the model found all positive instances that reflected the relation between the same entity pair at the sentence level, then constructed a combined sentence vector to fully utilize the semantic information of positive instances, achieving higher accuracy than the compared model. Yuxin et al.^18^ hypothesized that “the label of the final sentence alignment is a noisy observation result generated based on some unknown factors.” They learned the transition probability from noisy labels to true labels by training on automatically labeled data for relation extraction, achieving better results than mainstream baseline models. From a focus perspective, the sentence-level attention mechanism considers the overall context of a sentence, while the bag-level attention mechanism focuses on multiple sentences within a bag. Both mechanisms may encounter interference from irrelevant internal information when processing sentences. In terms of computational complexity, both the sentence-level and bag-level attention mechanisms may result in higher computational complexity when dealing with longer text sequences. To some extent, both mechanisms can improve the performance of the relation extraction task. However, further enhancing the model’s performance could be achieved by identifying the position of finer-grained semantic information that contributes more to the relation extraction task and allocating more attention to that specific segment.

If there is too much noise interference within the positive corpus, the corpus may be considered false positive by the program due to its low weight after attention calculation. This is catastrophic for distant supervision datasets with a large number of noisy sentences.

To accurately identify the relationship between two entities in a sentence, we need to focus on the semantic information within the sentence. A complete sentence typically consists of components such as subject, predicate, object, and adverbial. If a sentence can semantically express the relationship between two entities, it must be related to the key semantic information in the sentence, while other information is considered irrelevant or interfering noise. Liu et al.’s study^19^ showed that in the classic dataset of distant supervised relation extraction, NYT-Freebase, nearly 99.4% of sentences contain a large amount of noisy words. If the entire sentence is input into the model for training without processing the fine-grained semantic features, it will inevitably be affected by irrelevant noise within the sentence, thus affecting the overall performance of the model.

This paper proposes a remote supervision relationship extraction model based on fine-grained semantic information piecewise convolutional neural networks (PCNN + FGSI). The main contributions of this paper are as follows: (1) a new intra-sentence attention mechanism is proposed, which is different from the coarse-grained attention mechanism established at the sentence level. It is used to process fine-grained semantic features within the sentence, highlighting key semantic information and preventing irrelevant information and noise information from participating in the construction of sentence feature vectors with the same weight; (2) Based on (1), after obtaining sentence features that highlight fine-grained semantic information, a bag-level attention mechanism is used to screen positive training sentences with threshold gates and discard noisy sentences, in order to better distinguish positive and negative instances within all sentences containing the same entity pair and construct a combination feature vector to train the relationship classification network; (3) Comparative experiments and ablation experiments are designed to verify the performance advantages of the proposed relationship extraction method.

## Segmented convolutional neural network models based on fine-grained semantic information

**Figure 1:**
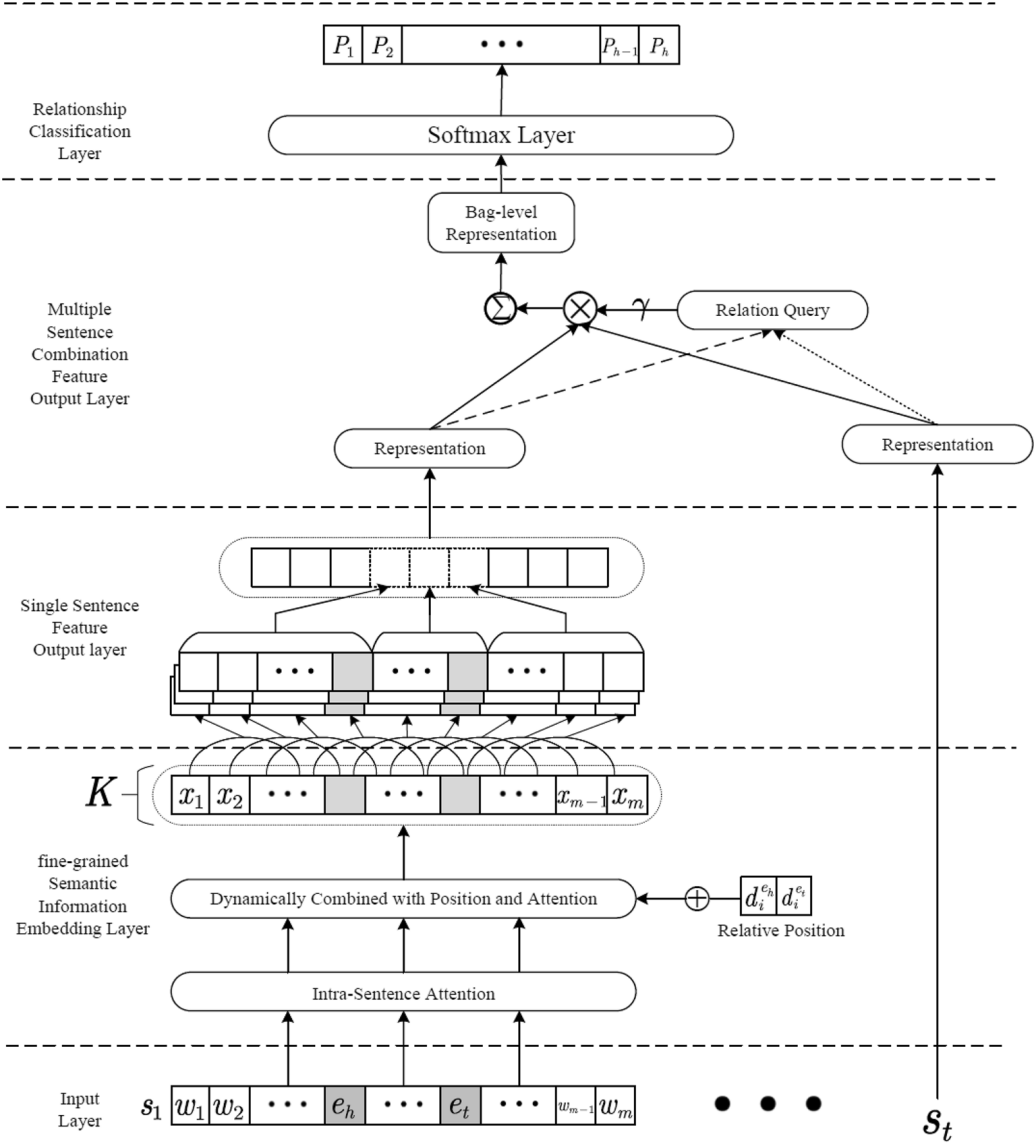
Overall architecture of the PCNN + FGSI model.

This paper proposes a fine-grained semantic information piecewise convolutional neural network model (**PCNN + FGSI**) for remote supervised relation extraction. The entire model consists of four parts, namely the text embedding layer based on fine-grained semantic information, the single-sentence feature output layer, the multi-sentence combined feature output layer, and the relation classification layer. The overall structure of the model is shown in Fig. 1.

In the text embedding layer based on fine-grained semantic information, the entire sentence is divided into three parts based on the positions of the two entities, and then the intra-sentence attention mechanism is applied to increase the weight of the part containing key semantic information and decrease the weight of the part containing noise information. The resulting representation emphasizes fine-grained semantic information. After obtaining the semantic embedding representation that emphasizes fine-grained semantic information, the single-sentence feature representation is formed through the encoding layer. The package-level attention mechanism in the multi-sentence combined feature output layer is used to screen positive instance information from the sentence feature representations containing the same entity pair. The weights of the positive instance feature representations are obtained and then the feature vectors are recombined. The recombined feature vectors are sent to the relationship classification layer to train the classifier, which improves the training performance of the model.

### Text embedding layer based on fine-grained semantic information

**Figure 2:**
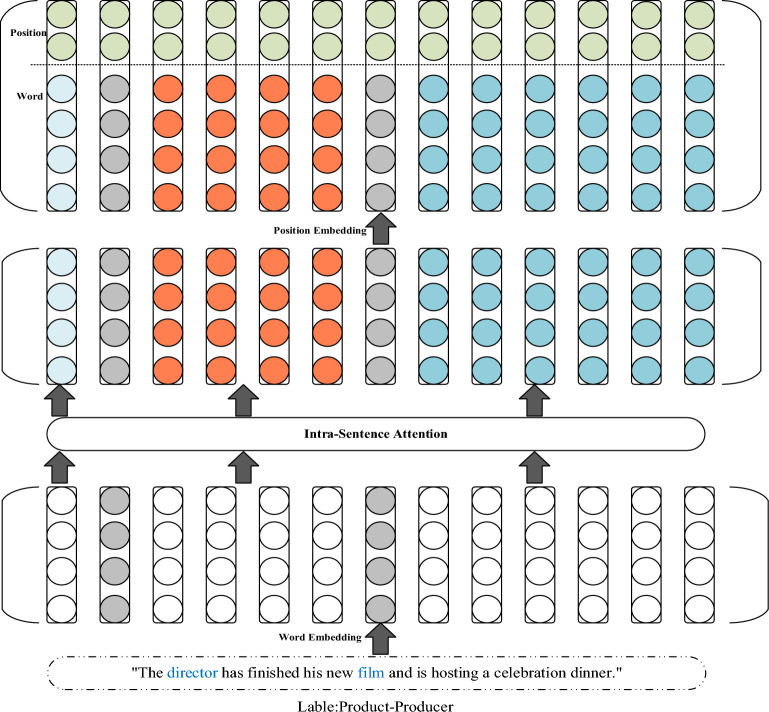
Structure diagram of the text embedding layer based on fine-grained semantic information.

The proposed model relies on neural networks to accomplish the task of relation extraction. However, natural language text cannot be directly used by neural networks. Therefore, when completing natural language processing tasks with neural networks, the first step is to convert the natural language text into a real-valued vector representation. The based on fine-grained semantic information text embedding layer of this model processes natural language text in three steps, namely word embedding, intra-sentence attention mechanism, and relative position information embedding. The structure of the based on fine-grained semantic information text embedding layer is shown in Fig. 2. After the training corpus is embedded by the word embedding part, the key semantic information part is given a greater weight by the intra-sentence attention mechanism, and then the relative position embedding information is concatenated to form the embedding vector representation of the sentence.

#### Word embedding

**Figure 3:**
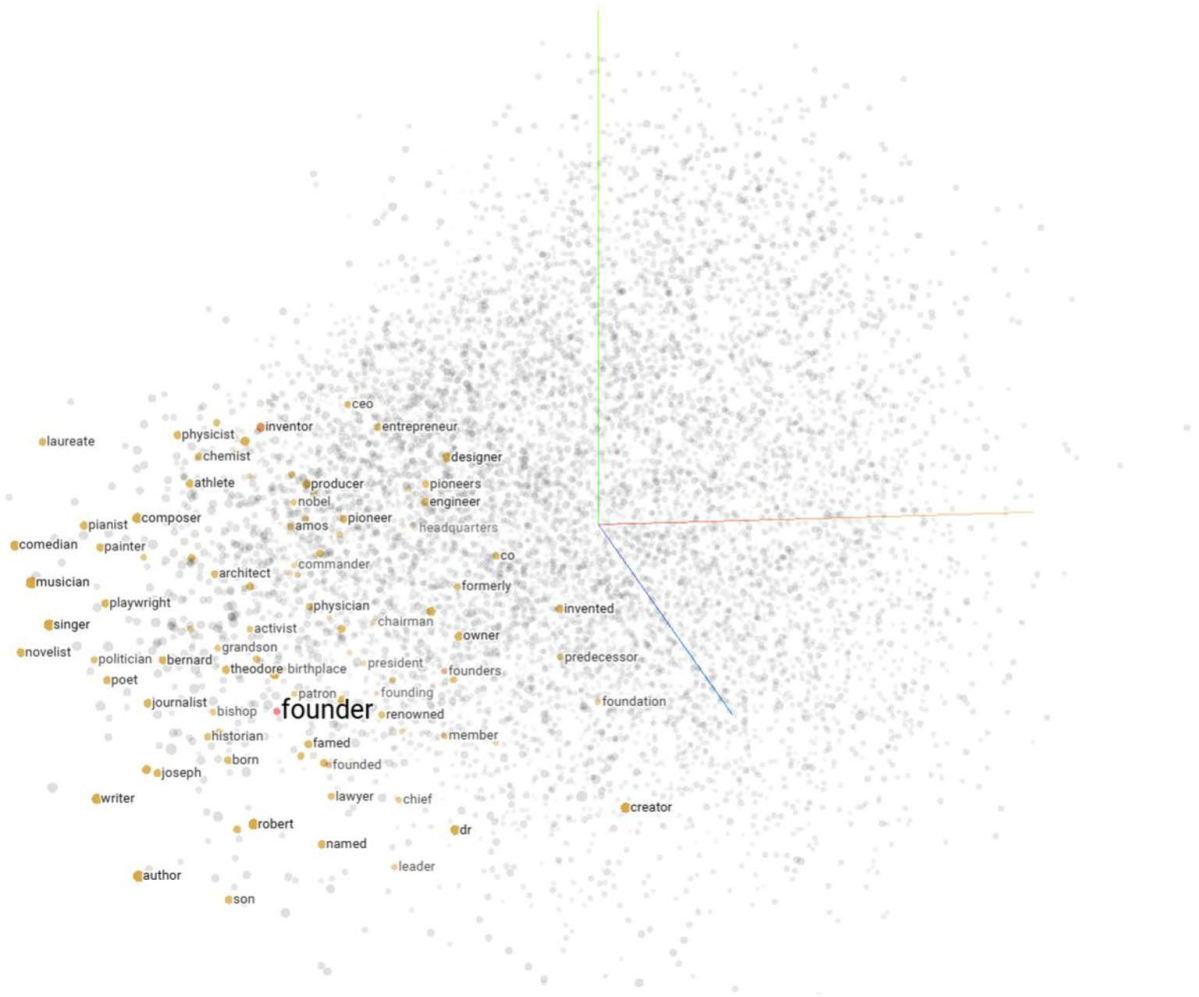
Distribution of semantics in space.

Word embedding is the process of transforming words into computable vectors, which are low-dimensional distributed representations of each word. The effectiveness of word embeddings in many natural language processing tasks has been demonstrated by Socher et al.^20^. Different methods have been proposed to train word embeddings, such as those by Bengio et al.^21^ and Mikolov et al.^22^. Currently, the most commonly used pre-trained word vectors are LSA (Latent Semantic Analysis), Word2vec, and GloVe. LSA is an early count-based word vector representation tool based on co-occurrence matrix. It uses matrix factorization techniques based on singular value decomposition (SVD) to reduce dimensionality of large matrices. However, the computational cost of SVD is high. Word2vec’s major limitation is that it only utilizes the corpus within a fixed window and does not fully leverage all the available corpus. GloVe combines the advantages of both methods. Figure 3 shows the distribution of the top 100 words with cosine similarity to the word “founder” in the semantic space of GloVe.

In this model, we use the pre-trained word embeddings method from Stanford GloVe. Given a sentence $s = \left( {w_{1} ,w_{2} ,w_{3} ,e_{{\text{h}}} ,w_{5} , \ldots ,w_{l} ,e_{{\text{t}}} ,w_{l + 2} , \ldots ,w_{m} } \right)$, each word is represented as a ${k}_{w}$-dimensional real-valued vector using the pre-trained word embedding matrix ${\varvec{E}}\in {{\varvec{R}}}^{\left|V\right|\times {k}_{w}}$, where ${e}_{h}$ and ${e}_{t}$ represent the head and tail entities, respectively. V is the size of the vocabulary (the number of words in the pre-trained word embedding corpus), and $m$ is the length of the sentence.

#### Intra-sentence attention mechanism

Assuming a sentence $s = \left( {w_{1} ,w_{2} ,w_{3} ,e_{h} ,w_{4} , \ldots ,w_{l} ,e_{t} ,w_{l + 2} , \ldots ,w_{m} } \right)$ contains an entity pair $\left\langle {e_{h} ,e_{t} } \right\rangle$ and is labeled with relation $r$, the word embedding vector representation ${s}{\prime}$ of the sentence can be obtained using “Word embedding” section, which is a matrix ${{\varvec{W}}}^{m\times {k}_{w}}$, where $m$ is the number of words in the sentence and ${k}_{w}$ is the dimension of the word embedding. In this paper, the word embedding vector representation of the sentence ${s}{\prime}=\left\{{s}_{1}{\prime},{s}_{2}{\prime},{s}_{3}{\prime}\right\}$ is divided into three segments based on the positions of the two entities $<{e}_{h},{e}_{t}>$ in the sentence. If a sentence can express the semantic relationship between its two internal entities, it must be related to key semantic information. After dividing the sentence into three parts according to the positions of the entities, the contributions of different parts to the model’s ability to extract the correct entity relation are different. To enable the model to better understand the key semantic information that expresses different entity relations, different weights are assigned to these three parts to reflect their contribution to relation r. The equation for calculating the weight of each part is as follows:

1 $$\alpha_{i} = \frac{{{\text{exp}}\left( {e_{i} } \right)}}{{\mathop \sum \nolimits_{k = 1}^{3} {\text{exp}}\left( {e_{k} } \right)}},1 \le i \le 3$$

where ${e}_{i}$ is the contribution of the i-th segment of the sentence to the relation label $r$ after the sentence is divided into three parts, and the calculation equation is as follows:

2 $$e_{i} = \frac{{s_{i}^{\prime } \cdot r^{\prime } }}{{s_{i}^{\prime } \times r^{\prime } }} = \frac{{\mathop \sum \nolimits_{j = 1}^{{k_{w} }} s_{ij}^{\prime } \times r_{j}^{\prime } }}{{\sqrt {\mathop \sum \nolimits_{j = 1}^{{k_{w} }} \left( {s_{ij}^{\prime } } \right)^{2} } \times \sqrt {\mathop \sum \nolimits_{j = 1}^{{k_{w} }} \left( {r_{j}^{\prime } } \right)^{2} } }}$$

where ${s}_{i}{\prime}$ represents the embedded vector representation of the $i$-th part of the sentence after embedding, and ${r}{\prime}$ represents the embedded vector representation of the relationship label $r$ in the semantic space used by this model. After calculating the contribution of each part, the formula for calculating the final embedded vector of the sentence is as follows:

3 $$s^{\prime} = \left[ {a_{1} s_{1}^{\prime } ;\;a_{2} s_{2}^{\prime } ;\,a_{3} s_{3}^{\prime } } \right]$$

#### Position embedding

**Figure 4:**
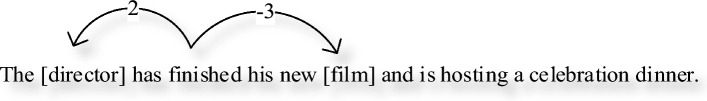
Example of relative distance.

Zeng et al.^10^ have shown through experiments the importance of positional features in relation extraction tasks. Feng et al.^17^ also argue that when judging the relationship between entity pairs in a sentence, words that are closer to the entities are usually key information. Therefore, in order to better capture the structural information of a sentence, this paper introduces positional embeddings in the embedding stage, using positional features to record the relative distances of each word to the two entities. An example of relative distances is shown in Fig. 4.

The model looks up the relative distance of each word ${\mathrm{w}}_{\mathrm{i}}$ to the two entities, and then maps these two relative distances to two ${k}_{p}$-dimensional real-valued vectors $({\mathrm{d}}_{\mathrm{i}}^{{\mathrm{e}}_{\mathrm{h}}},{\mathrm{d}}_{\mathrm{i}}^{{\mathrm{e}}_{\mathrm{t}}})$. For each sentence that needs to be trained in the model, its word embedding and position embedding are concatenated to obtain the sentence vector representation matrix ${\varvec{X}}=[{x}_{1},{x}_{2},\cdots ,{x}_{m}]\in {{\varvec{R}}}^{m\times k}$, where ${{\varvec{x}}}_{i}=[{w}_{i};{\mathrm{d}}_{\mathrm{i}}^{{\mathrm{e}}_{\mathrm{h}}};{\mathrm{d}}_{\mathrm{i}}^{{\mathrm{e}}_{\mathrm{t}}}]$, $m$ denotes the length of the sentence, $k$ is the dimension after concatenating the word embedding and position embedding vectors, that is, $k= {k}_{w}+{k}_{p}\times 2$.

### Single-sentence feature output layer

**Figure 5:**
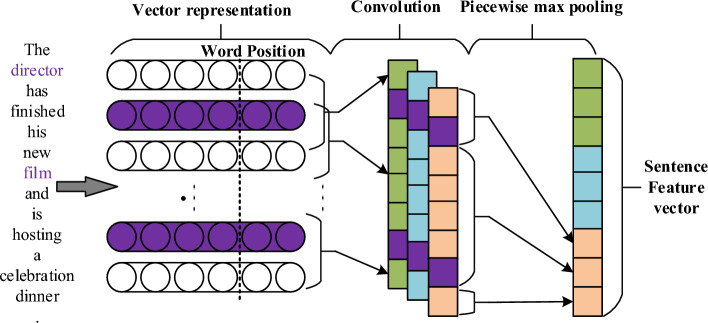
Network structure of PCNN.

The effectiveness of the PCNN model for sentence-level feature extraction has been demonstrated in the studies by Zeng et al.^10^ and G. Ji et al.^15^. Therefore, in this paper, we adopt the PCNN structure as the single-sentence feature output layer of our model, as shown in Fig. 5. After obtaining the embedded representation of the sentence, the embedding vector is fed into the PCNN structure, and the sentence’s feature vector representation is obtained through convolutional and piecewise max-pooling computations.

#### Convolution

In the task of entity relation extraction, the length of each sentence varies. To address this issue, sentence padding is applied to align the length of the corpus. The alignment standard is based on the longest sentence in each $batch$ of samples. Additionally, effective information for determining the relationship between target entities may exist at different positions within a sentence. To capture such information from different positions, the model needs to extract local features at different scales to predict the relationship classification for the entity pair. Dumoulin et al.^23^ conducted in-depth research on convolution algorithms in deep learning. In deep learning, the convolution operation is often used to extract local features of different scales.

After the text is embedded with the fine-grained semantic information in the text embedding layer, the final embedding vector representation of the input sentence is defined as $s^{\prime \prime } = \left\{ {b_{1} ,b_{2} , \ldots ,b_{{\left| {s^{\prime \prime } } \right|}} } \right\}$, where $b_{i}$ denotes the embedding vector representation of the $i$-th word in the sentence, and ${b}_{i}\in {{\varvec{R}}}^{\left|k\right|}$. In this paper, ${s}_{i:j}^{{\prime}{\prime}}$ is used to represent the horizontal concatenation matrix of the embedding sequence $\left[ {b_{i} ,b_{i + 1} , \ldots ,b_{j} } \right]$ in the sentence, and $w$ represents the length of the filter operator. The weight matrix of the filter operator is denoted as ${\varvec{W}}\epsilon {{\varvec{R}}}^{w\times k}$. The convolution operation is performed by filtering the embedding vector representation of the sentence with the filter operator, and a vector $c\in {R}^{\left|{s}^{{\prime}{\prime}}\right|-w+1}$ is obtained, as shown in Eq. (4):

4 $$c_{j} = {\varvec{W}} \otimes s_{{\left( {j - w + 1} \right):j}}^{\prime \prime }$$

In this formula, $1\le j\le \left|{s}^{{\prime}{\prime}}\right|-w+1$. During the feature extraction process through convolution, different filter kernels are needed to extract feature information at various positions in the sentence instance. Therefore, $n$ different filter kernels are used, and correspondingly, there are $n$ weight matrices $\hat{\user2{W}} = \left\{ {{\varvec{W}}_{1} ,{\varvec{W}}_{2} , \ldots ,{\varvec{W}}_{n} } \right\}$. All convolution operations during the feature extraction process can be represented by Eq. (5):

5 $$c_{ij} = {\varvec{W}}_{i} \otimes s_{{\left( {j - w + 1} \right):j}}^{\prime \prime }$$

Here, $1\le i\le n$ and $1\le j\le \left|{s}^{{\prime}{\prime}}\right|-w+1$. The convolution operation produces feature vectors for each sentence, denoted as $C = \left\{ {c_{1} ,c_{2} , \ldots ,c_{n} } \right\}$.

#### Piecewise max pooling

After the convolution operation in “Convolution”, the feature vector ${c}_{i}$ can be obtained, which can be represented as ${c}_{i}=\left\{{c}_{i\_1},{c}_{i\_2},{c}_{i\_3}\right\}$ by dividing the sentence instance into three parts according to the positions of the given entities. Based on this vector, the segmented max pooling operation is performed, i.e., ${p}_{ij}=\mathrm{max}({c}_{i\_j})$, where $1\le i\le n, j=\mathrm{1,2},3$. Then, the resulting vectors are concatenated to obtain ${p}_{i}=\left[{p}_{i1},{p}_{i2},{p}_{i3}\right](i=\mathrm{1,2},\dots ,n)$, where $p\epsilon {{\varvec{R}}}^{3n}$. This represents the feature vector of each sentence obtained after being processed by the PCNN structure.

### Multilingual sentence combination feature output layer

**Figure 6:**
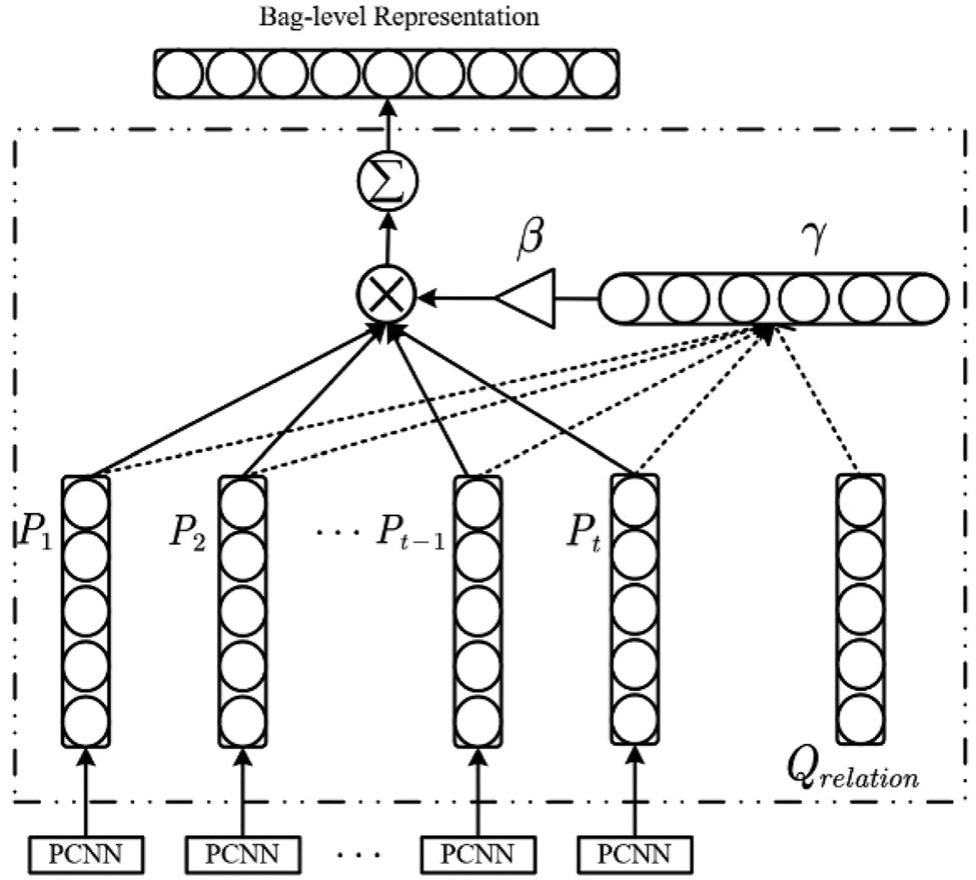
Multilingual sentence combination feature output layer.

In order to automatically filter out noisy sentences with significant differences from the labels in the task of remote supervised relation extraction, this layer adopts a multi-instance learning strategy and an intra-bag attention mechanism. It filters out low-relevant sentences within bags through a threshold gate after attention calculation and combines the features of all positive instances to form the training vector for the final classifier. The structure of this layer is shown in Fig. 6. Using the associated query vector, attention calculation is performed on each sentence feature vector within the bag, resulting in corresponding weights. Sentences with weights lower than the hyperparameter β are filtered out using a threshold gate, forming a bag-level vector representation, which is then inputted into the classification layer for training.

This paper includes all sentences that contain the given entity pair $\langle {e}_{1},{e}_{2}\rangle$ and have a relationship label $r$ into the set $S$. Assuming that there are $t$ sentences that meet the requirement, the set $S$ can be represented as $S=\left\{{s}_{1},{s}_{2},{s}_{3},\dots ,{s}_{t}\right\}$. After obtaining the feature vector representation $p$ for each sentence in “Single-sentence feature output layer” section, the vector set P corresponding to the sentence set $S$ can be represented as $P=\left\{{p}_{1},{p}_{2},{p}_{3},\dots ,{p}_{t}\right\}$. Due to the noise problem in remote supervision, each sentence in this set expresses the relationship label $r$ differently. Therefore, an intra-bag attention mechanism is adopted to set a weight that can express the relationship label $r$ for each sentence through attention calculation. After filtering out low-relevant sentences using the threshold gate, the weights $\left({\gamma }_{1},{\gamma }_{2},{\gamma }_{3},\dots ,{\gamma }_{n}\right)$ calculation formula for the sentences that contribute to the formation of the bag-level vector representation is shown as Formula (6):

6 $$\gamma_{i} = \frac{{{\text{exp}}\left( {e_{i} } \right)}}{{\mathop \sum \nolimits_{k = 1}^{n} {\text{exp}}\left( {e_{k} } \right)}},1 \le i \le n;e_{i} \ge \beta$$

Here, ${e}_{i}$ represents the relevance degree of the i-th sentence in the set $S$ to the relationship label $r$, and its calculation formula is shown in Eq. (7):

7 $$e_{i} = \frac{{p_{i} \cdot Q_{relation} }}{{\parallel p_{i} \parallel \times \parallel Q_{relation} \parallel }},1 \le i \le t$$

Here, ${p}_{i}$ represents the feature vector of the i-th sentence in the sentence set $S$, and ${Q}_{relation}$ is the vector representation of the relationship label $r$ in the semantic space, representing the weight of the relationship label $r$ in calculating each sentence.

After the calculation of intra-sentence attention, each sentence in the set $S$ has obtained a weight that expresses the relationship label $r$. This paper believes that different sentences in the same set have different degrees of expression for the relationship label $r$, which can be reflected in the weight $\gamma$ accordingly. Therefore, positive instances score high on weight $\gamma$, while negative instances score low on weight $\gamma$. Based on the above assumptions, by setting the hyperparameter $\beta$, when forming the combination feature vector of multiple sentences, the sentence vectors with weights lower than $\beta$ are filtered out, thus avoiding noise sentences from participating in the formation of combination feature vectors with low weights. Assuming that after filtering out noise sentences, there are still $n$ sentences left in the set $S$, the formula for generating the combination feature vector of the set is shown in Eq. (8):

8 $$g = \mathop \sum \limits_{j = 1}^{n} \gamma_{i} p_{j} ,1 \le j \le n$$

### Relation classification layer

For the set $S$ in “Multilingual sentence combination feature output layer” section, where the distant supervision relationship label is known, in order to compute the probability distribution of the combined feature vector of the set for relationship classification, the $softmax$ layer is applied to the relationship classification layer in this paper. Assuming that the combined feature vector of the $i$-th set $S$ is denoted as ${g}_{i}$, the probability distribution of the relationship obtained by passing the combined feature vector through the softmax layer is shown in Eq. (9):

9 $$P\left( {r_{i} |g_{i} } \right) = softmax\left( {{\varvec{W}}_{o} g_{i} + b_{o} } \right)$$

Here, ${{\varvec{W}}}_{{\varvec{o}}}\epsilon {{\varvec{R}}}^{h\times 3n}$, where $h$ represents the number of pre-defined relations.

### Optimization

The model parameters to be optimized in this paper are $\theta =(E,{D}_{he1}, {D}_{te2},W,{W}_{o})$, where $E$ represents the word embeddings, ${D}_{he1}$ represents the position vectors of words relative to the head entity, ${D}_{te2}$ represents the position vectors of words relative to the tail entity, $W$ represents the parameters involved in the convolutional operation, and ${W}_{o}$ represents the parameters of the relation classification layer. The cross-entropy loss function used in this model is defined as shown in Eq. (10):

10 $$J\left( \theta \right) = \mathop \sum \limits_{i = 1}^{N} \log p(r_{i} |g_{i} ,\theta )$$

where $N$ is the number of sentence sets, and ${g}_{i}$ represents the combined feature vector of the $i$-th sentence set.

During parameter updates, Li et al.^24^ compared four common optimizers by performing parameter optimization on the hand-written digit recognition MNIST dataset and the FASHION dataset. Among them, the $Adam$ optimizer performed well. Therefore, the $Adam$ optimizer was used as the parameter update optimizer for the model in this paper. The Adam optimizer combines the first-order moment of the gradient of SGD-M and the second-order moment of the gradient of RMSprop, taking into account the mean and variance of the gradient, and adds two correction terms on this basis. The formula is shown in Eqs. (11)–(13):

11 $$m_{t}^{1} = \frac{{m_{t} }}{{1 - \beta_{1}^{\tau } }}$$

12 $$v_{t}^{2} = \frac{{v_{t} }}{{1 - \beta_{2}^{\tau } }}$$

13 $$\omega_{t + 1} = \omega_{t} - lr \times \frac{{m_{t}^{1} }}{{v_{t}^{2} }}$$

Here, ${m}_{t}^{1}$ represents the bias-corrected first moment estimate and ${v}_{t}^{2}$ represents the bias-corrected second moment estimate, where ${\beta }_{1},{\beta }_{2}\in \left[\mathrm{0,1}\right]$ are the decay rates of the first and second moment estimates respectively, and $lr$ denotes the learning rate.

## Experimentation and evaluation

To demonstrate the effectiveness of the proposed method in this paper, comparative experiments and ablation experiments were designed in this section to demonstrate the advantages of the proposed method from different perspectives.

### Dataset and evaluation metrics

**Figure 7:**
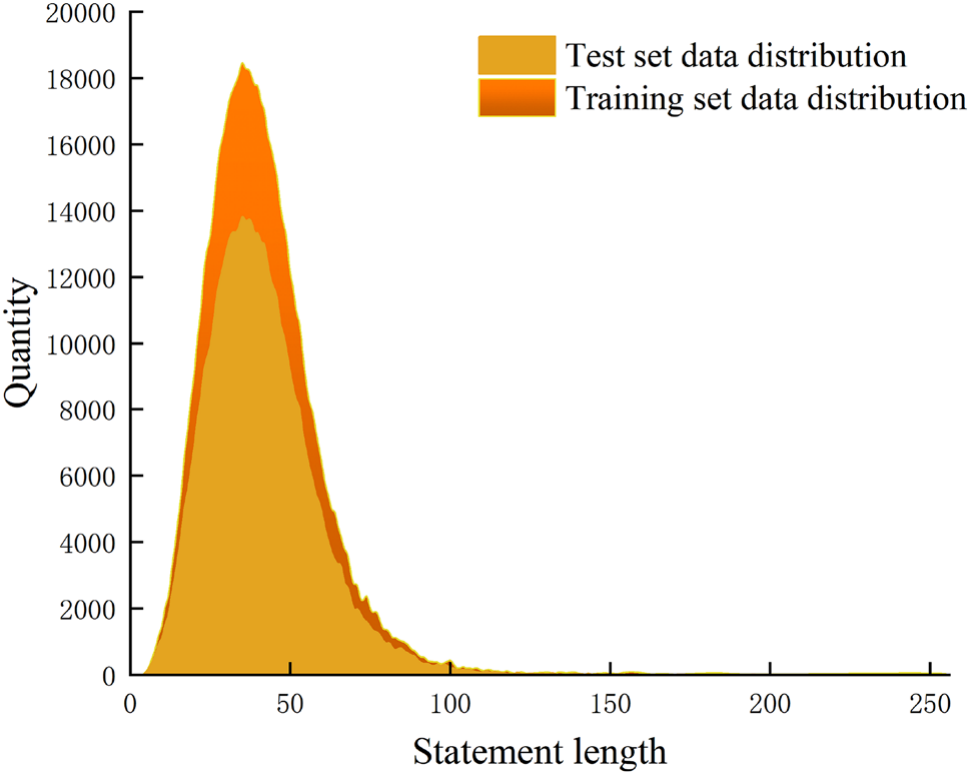
Data distribution of NYT-10 dataset.

The NYT-10 dataset was released by Riedel et al.^12^, and many domestic and foreign scholars have conducted research on distant supervision relation extraction based on this dataset^25,26^. The dataset is aligned with relations in Freebase, and the sentences obtained from news corpus from 2005 to 2006 are used as the training set, while the sentences obtained from news corpus in 2007 are used as the test set. The dataset contains 53 types of relations, including the special relation type “NA”, which indicates that there is no relation between two entities. In both the training and test sets, the special relation type “NA” has the largest proportion among all the training sentences. We set the maximum length of sentences in the dataset to 256, and Fig. 7 shows the distribution of sentence lengths in the NYT-10 dataset. It can be seen that the maximum length of sentences is concentrated within [20, 60].

We use the held-out evaluation method to evaluate the proposed relation extraction model, and evaluate the performance of the model through the $PR\left(Precision-Recall\right)$ curve and $\mathrm{P}@\mathrm{N}(\mathrm{Precision}@\mathrm{Top N})$.

### Parameter settings

In this study, we tested the performance of the model on the test dataset by adjusting parameters such as the maximum length of training sentences, polynomial decay learning rate, hyperparameters, and batch size. The other parameters were the same as those used by Lin et al.^27^. Table 1 shows the main parameters used in the experiments of this study.

**Table 1:** Parameter settings.

Parameter description	Configuration
Convolutional kernel size	3, 4, 5
Number of convolutional kernels	200
Word embedding dimension	200
Positional embedding dimension	5
Batch Size	128
Dropout	0.5
β	0.25
Initial learning rate minimum learning rate	1E-2
Initial learning rate minimum learning rate	1E-6

### Comparative experimental results and analysis

To evaluate the proposed method on the NYT-10 dataset, we selected several classic baseline methods for comparison through held-out evaluation. The compared baseline methods are:

- Mintz^6^: Mintz first proposed the idea of distant supervision and combined the advantages of supervised and unsupervised information extraction.
- MultiR^28^: This model, proposed by Hoffmann et al., combines a sentence-level extraction model with a simple corpus-level component for aggregating single facts.
- MIML^8^: This is a multi-instance multi-label learning method proposed by Surdeanu.
- PCNN + MAX^10^: This method, proposed by Zeng, trains instances with the maximum logistic regression value.
- PCNN + ATT (Sentence-level Selective Attention Model)^27^: This is an improved model based on the PCNN model, proposed by Lin et al., which uses sentence-level attention mechanism.
- PCNN + MIL^10^: This method, proposed by Zeng, combines the advantages of multi-instance learning and the PCNN model.
- PCNN + RL^29^: This method, proposed by Feng et al., applies reinforcement learning to instance selectors to choose high-quality sentences for training the relation classifier.
- APCNNS^15^: This is an extraction method that combines PCNN with entity information, proposed by Ji.
- BGWA^30^: This method, proposed by Jat et al., uses word-level attention mechanism for relation extraction tasks.
- PCNN + ATT + N^18^: Combining the noisy observation model with deep neural networks, the research focuses on the noise distribution attention mechanism and denoising methods for imbalanced samples.
- BERT + GCN^31^: An external knowledge enhancement module has been added to the existing model, which preprocesses and encodes the existing entity types and relations in the knowledge base. This provides the model with external knowledge that is not present in the sentence-level text.
- PARE^32^: This method improves the performance of the relation extraction task by integrating position feature attention mechanism and relation enhancement mechanism.
- PCNN + BATT^33^: This method proposes the combination of intra-bag attention mechanism and inter-bag attention mechanism for distantly supervised relation extraction task.

**Figure 8:**
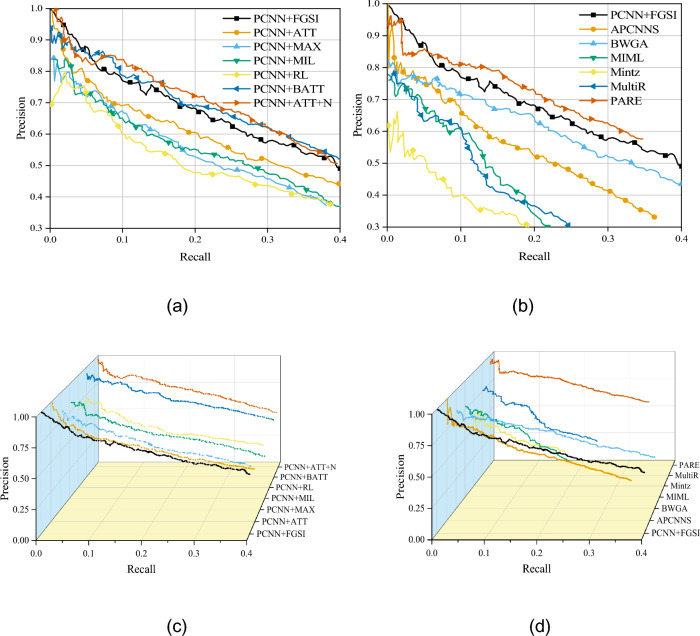
Precision-recall curve.

From Fig. 8a,c, it can be observed that in the PCNN-based improved model, our proposed PCNN + FGSI model maintains good performance across the entire recall range. It outperforms other models in the recall range of 0–0.5. Compared to other PCNN-based improved models, our model is able to locate the position of key semantic segments in the sentence through attention mechanism. It assigns higher weights to these segments to contribute to the composition of sentence vectors. Additionally, when dealing with longer text sequences, our model effectively decomposes them for computation, reducing computational complexity. Furthermore, the intra-sentence attention mechanism proposed in this paper allows for local attention, effectively reducing interference from irrelevant information and improving the accuracy of entity relation recognition.

From Fig. 8b,d, it can be observed that in comparison with other classic models, our proposed method also exhibits excellent performance across the entire recall range. Our method divides the sentence based on the positions of entity pairs and calculates attention weights separately. This helps the model effectively align the semantic information of the entities and capture local semantic information between entity pairs, thereby enhancing the effectiveness of relation extraction.

Table 2 shows the comparison of P@N values between the proposed relation extraction method and baseline models. As can be seen from the table, among all the baseline models, the BGWA model has the slowest precision decline. Although the proposed PCNN + FGSI model does not perform as well as the BGWA model in terms of the rate of precision decline, it performs the best within the scope of the indicators. The average precision of PCNN + FGSI model is 8% points higher than that of the PCNN + ATT model, which further validates the advantages of the proposed method. The experimental results demonstrate that our distantly supervised relation extraction model outperforms other comparative models in terms of AUC value. This confirms the effectiveness and superiority of our model in relation extraction tasks.

**Table 2:** P@N、AUC comparison table of PCNN + FGSI and baseline model.

Methods	P@N (%)				AUC (%)
	100	200	300	Average	
Mintz	54.0	50.5	45.3	49.9	10.7
MIML	70.9	62.8	60.9	64.9	–
MultiR	64.0	61.5	53.7	59.7	–
PCNN + MAX	73.3	70.3	65.3	69.6	21.6
PCNN + ATT	81.1	71.1	69.4	73.9	34.1
PCNN + MIL	74.3	71.7	66.1	70.7	–
PCNN + RL	74.8	68.2	61.9	68.3	–
PCNN + BATT	76.9	75.4	72.9	75.1	35.1
APCNNS	76.3	74.2	69.4	73.3	–
BGWA	75.2	74.1	71.4	73.6	34.0
PARE	–	–	–	–	48.1
PCNN + FGSI	86.5	82.7	76.4	81.9	49.6

### Influence of threshold setting in intra-bag attention mechanism on model effect

In “Multilingual sentence combination feature output layer” section, this paper discusses the Multilingual Sentence Combination Feature Output Layer of the model. Additionally, improvements are made to the bag-level attention mechanism. By setting a threshold β, this paper filters out low-relevant sentences within a bag, preventing their participation in the composition of bag-level vector representations. This further reduces noise interference within the dataset.

The setting of the threshold β also has an impact on the performance of the relation extraction model. Therefore, further experimental analysis is conducted on this aspect, and the results are shown in Table 3.

**Table 3:** The effect of β value on model effect.

	P@N (%)			
	100	200	300	Average
β = 0.10	83.1	80.6	73.2	79.0
β = 0.15	83.5	80.9	73.6	79.3
β = 0.20	84.3	81.8	74.7	80.3
β = 0.25	**86.5**	**82.7**	**76.4**	**81.9**
β = 0.30	84.1	81.3	74.9	80.1
β = 0.35	83.5	80.7	74.2	79.5

Significant values are in bold.

As can be seen from Table 3, with the increase of threshold β, the performance of the model does not continue to increase. When threshold β reaches 0.25, the model achieves excellent results.

### Ablation experiment results and analysis

**Figure 9:**
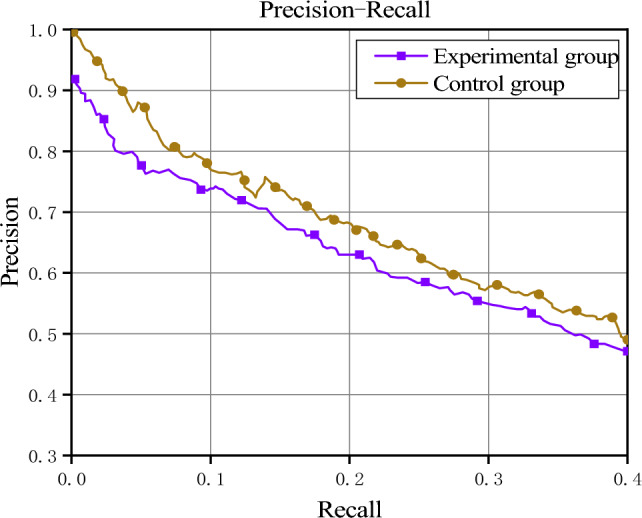
PR curve of the control experimental group.

In this article, a series of ablation experiments were designed in order to investigate the role of the fine-grained semantic information text embedding layer in model experiments. In this experiment, the control group (CG) represents the model proposed in this article (PCNN + FGSI), while the experimental group (EG) blocks the intra-sentence attention mechanism proposed in this article during the text embedding stage. Figure 9 depicts the precision-recall curves of the experimental group and the control group.

From Fig. 9, it can be seen that the control group performs the best on the PR curve. The performance of the experimental group drops slightly when using a regular text embedding layer. This is because the text embedding layer based on fine-grained semantic information can highlight the semantic information that expresses entity relationships in positive instances, enabling the model to learn fine-grained semantic information that expresses entity relationships, and thereby constructing more robust feature vectors.

This paper also uses P@N and AUC to compare the performance of the experimental group and the control group, as shown in Table 4.

**Table 4:** P@N comparison table of experimental group and control group.

Methods	P@N (%)				AUC (%)
	100	200	300	Average	
EG	82.3	78.4	74.2	78.3	44.1
CG	**86.5**	**82.7**	**76.4**	**81.9**	**49.6**

Significant values are in bold.

From Table 4, it can be observed that the experimental group with regular text embedding layer shows a decrease in performance in the P@N (N = 100/200/300) and AUC evaluation metrics compared to the control group. This is consistent with the conclusion obtained from the PR curve analysis, indicating that the text embedding layer based on fine-grained semantic information is helpful in improving the model performance.

## Conclusion

### Summary

This study aims to explore the issue of noisy data in the task of distant supervision for relation extraction. To address this problem, we propose a distant supervision relation extraction method based on fine-grained semantic information. This method segments sentences based on the positions of entity pairs. By utilizing intra-sentence attention mechanism, it effectively locates the positions of key semantic information segments within the sentences. Through attention calculation, greater weights are assigned to these key semantic information segments, constructing a sentence feature vector highlighting the key semantic information, and reducing interference from irrelevant information. Furthermore, this method improves the package-level attention mechanism and filters out low-relevant noisy sentences within a package through a threshold gate, further reducing the impact of noisy sentences on the model’s performance and making full use of existing positive semantic information. Experimental results on the NYT-10 dataset show that our method exhibits significant advantages in accuracy and other aspects compared to traditional methods.

### Prospect

The current research has considered the location information and entity pair description information, showing certain effectiveness in improving relation extraction models. In future research, we will consider selecting high-quality information that can express semantic relations from a linguistic perspective to participate in model training as external descriptive information. We will shift the research focus of relation extraction towards semantic studies, aiming to lay a certain research foundation and provide references for future researchers to conduct open-domain relation extraction. We believe that this study is of significant importance in addressing the issue of noisy data and promoting the development of the field of relation extraction.

## Data Availability

The data set used in this research work is publicly available and can be downloaded from the website below. **NYT-10:** OpenNRE/benchmark at master thunlp/OpenNRE (github.com).
